# Biomechanics of a cemented short stem: a comparative in vitro study regarding primary stability and maximum fracture load

**DOI:** 10.1007/s00402-021-03843-x

**Published:** 2021-03-23

**Authors:** Tobias Freitag, Karl Philipp Kutzner, Ralf Bieger, Heiko Reichel, Anita Ignatius, Lutz Dürselen

**Affiliations:** 1 Department of Orthopaedic Surgery, University of Ulm, Oberer Eselsberg 45, 89081 Ulm, Germany; 2 Department of Orthopaedic Surgery and Traumatology, St. Josefs Hospital Wiesbaden, Beethovenstr. 20, 65189 Wiesbaden, Germany; 3 Institute of Orthopaedic Research and Biomechanics, Trauma Research Centre Ulm, Ulm University, Medical Centre, Helmholtzstr. 14, 89081 Ulm, Germany

**Keywords:** Cemented total hip arthroplasty, Short stem, Biomechanics, Migration, Micromotion, Primary stability, Fracture pattern, Cadaver

## Abstract

**Purpose:**

In total hip arthroplasty, uncemented short stems have been used more and more frequently in recent years. Especially for short and curved femoral implants, bone-preserving and soft tissue-sparing properties are postulated. However, indication is limited to sufficient bone quality. At present, there are no curved short stems available which are based on cemented fixation.

**Methods:**

In this in vitro study, primary stability and maximum fracture load of a newly developed cemented short-stem implant was evaluated in comparison to an already well-established cemented conventional straight stem using six pairs of human cadaver femurs with minor bone quality. Primary stability, including reversible micromotion and irreversible migration, was assessed in a dynamic material-testing machine. Furthermore, a subsequent load-to-failure test revealed the periprosthetic fracture characteristics.

**Results:**

Reversible and irreversible micromotions showed no statistical difference between the two investigated stems. All short stems fractured under maximum load according to Vancouver type B3, whereas 4 out of 6 conventional stems suffered a periprosthetic fracture according to Vancouver type C. Mean fracture load of the short stems was 3062 N versus 3160 N for the conventional stems (*p* = 0.84).

**Conclusion:**

Primary stability of the cemented short stem was not negatively influenced compared to the cemented conventional stem and no significant difference in fracture load was observed. However, a clear difference in the fracture pattern has been identified.

## Introduction

Cemented total hip arthroplasty (THA) has a long history of success, being a safe strategy for the treatment especially for elderly patients with potentially reduced bone quality [1]. Registry data from Sweden, Norway and England show a better long-term survivorship of cemented compared to cementless implant fixation [2–4]. Data from the national registries in Australia and New Zealand characterize a lower revision rate of cemented compared to cementless stems, especially in female patients over 75 years [5, 6]. Femoral periprosthetic fractures following THA remain one of the leading causes of early failure requiring revision surgery [7–9]. In this regard, the main risk factors are reduced bone quality, advanced age and female gender [8, 10]. Consequently, cemented femoral stem fixation is strongly correlated with a decreased risk of early periprosthetic fractures of the femur, particularly in female and elderly populations [11, 12].

In the last decade, there has been a trend towards the development of shorter cementless femoral implants, aiming to enable a more bone- and soft tissue-sparing implantation technique [13–15]. Most implants of the latest generation provide a curved stem design, which allows the implantation without compromising the trochanteric region and thus the pelvitrochanteric structures. Promising medium- and long-term term data already exist for several shorter cementless implant models [16, 17]. Some authors confirm advantageous results regarding perioperative blood loss and a lower intraoperative complication rate compared to standard implants [18–20]. On the other hand, some authors propagate a limitation of this implant group, especially in poor bone quality, due to the shorter and ostensibly metaphyseal fixation [21, 22]. A markedly reduced bone quality was seen to be associated with a dramatically increased risk for postoperative periprosthetic femoral fractures using a cementless calcar-guided short stem [23]. Taking this contraindication into account, implant survival rates up to 100% at 8 years have been reported [24].

Currently, efforts are being made to transfer the postulated potential advantages of uncemented short-stem THA to the concept of a cemented short stem, providing the same philosophy, to extend the range of indications to a patient collective with reduced bone quality [25]. To date, no curved short stem, providing cemented fixation, is officially available on the market.

The aim of this in vitro study was to compare primary stability and fracture load of a newly developed, cemented curved short stem with an already well-established cemented conventional stem [26].

## Methods

### Implants

**Fig. 1:**
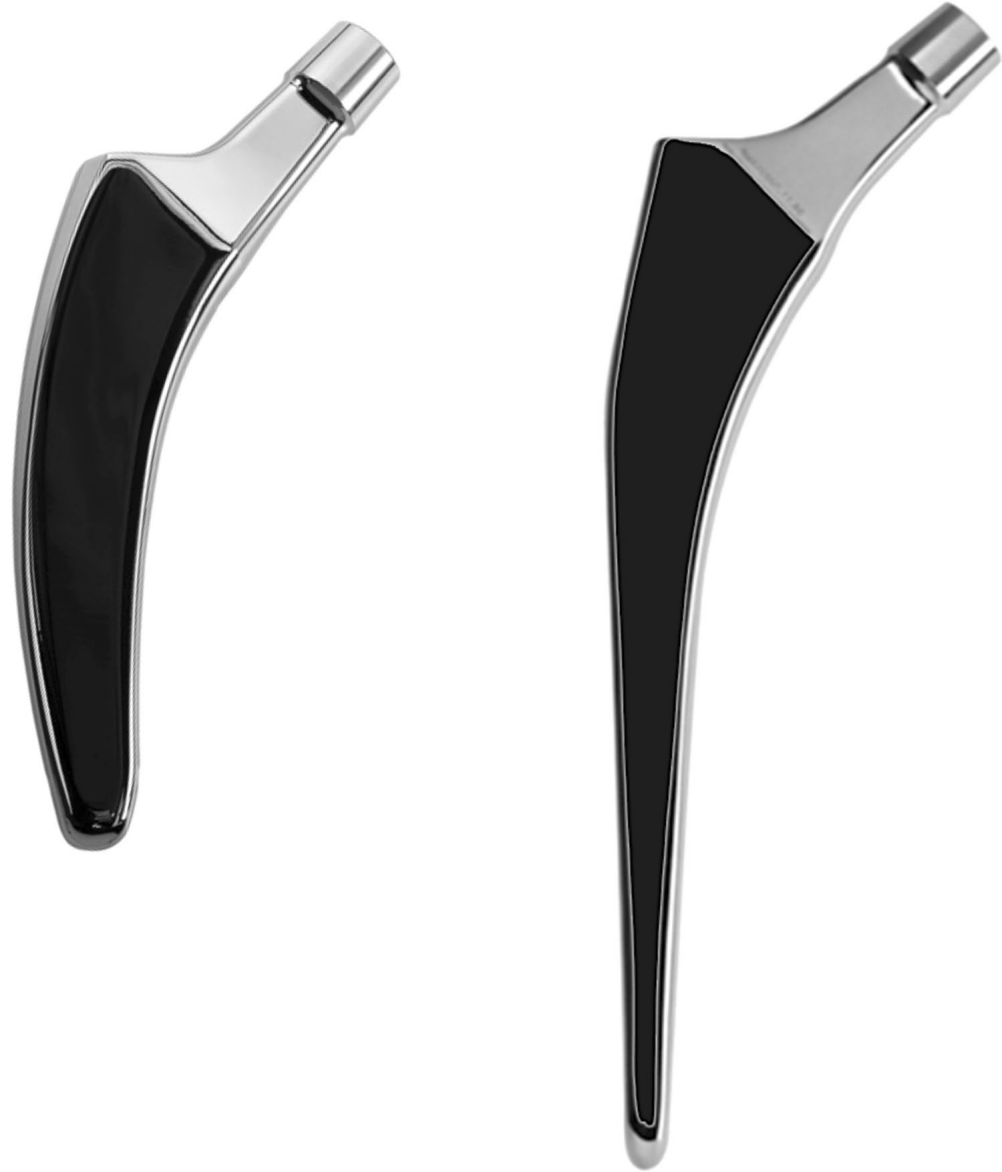
Cemented optimys (left), and twinSys stem (Mathys Ltd., Bettlach, Switzerland)

The prototype of the cemented optimys short stem (Fig. 1) is based on the design of the uncemented implant, which is available on the market since 2010 (optimys, Mathys Ltd., Bettlach, Switzerland). According to the concept of many successful cemented conventional stems on the market, the prototype is made of polished wrought high nitrogen stainless steel for implants based on ISO 5832-1. With 13 selectable sizes the stem length is between 80 and 118 mm. As a reference, the well-established cemented conventional straight twinSys stem (ODEP 7A*) was used in this study (twinSys, Mathys Ltd., Bettlach, Switzerland). The stem is available in 8 sizes with lengths between 140 and 170 mm (Fig. 1).

Both implants provide a triple taper design, which converts shear forces into compression forces and thus allows the stem to wedge into the cement mantle. Two different offset versions are available for both implants, standard and lateralized, for offering a broad offset range to reconstruct the individual femoral offset. An earlier study of our group showed no inferiority in terms of primary stability and maximum fracture load of the cemented short-stem prototype, using a line-to-line cementation technique, compared to a standard technique using an undersized stem [25]. Thus, a line-to-line cementation technique was used. In contrast, the design of the conventional stem used for this study, is undersized by 1 mm compared to the final rasp and thus, offers a minimal space for a preferably homogeneous cement mantle.

### Preparation of cadaver femurs

**Fig. 2:**
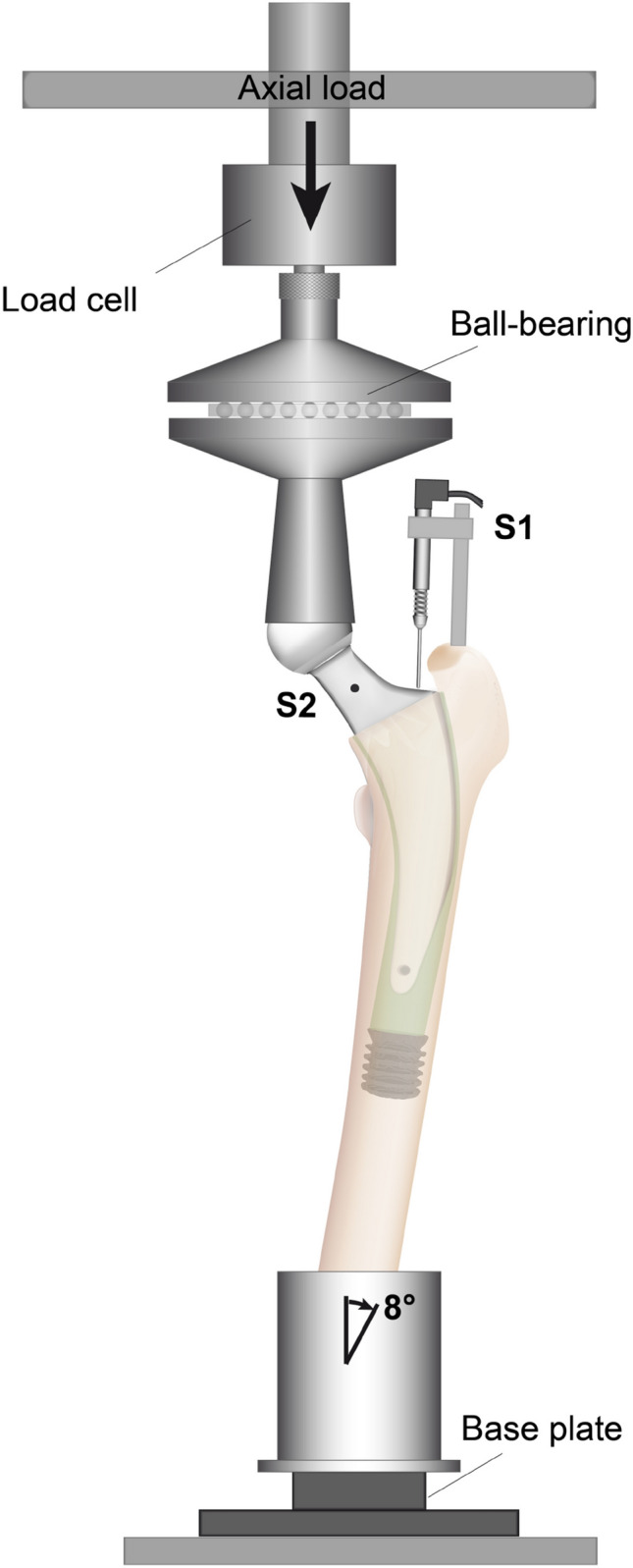
The test setup. S1 and S2 demonstrate the locations of the two miniature displacement transducers

After institutional review board approval, six osteoporotic pairs of fresh–frozen human femurs were obtained via ScienceCare (Phoenix, AZ, USA). All donors were female, with a mean age of 71 years (range 63–81 years) and a mean Body Mass Index (BMI) of 30.2 kg/m^2^ (range 18.9–42.6 kg/m^2^) (Table 1). Radiographs in two planes ruled out any malignant neoplasia or fractures. Digital 2D templating, using the original stem templates, estimated the size and positioning of the required femoral implants as well as the height of the neck resection. Minor bone quality was confirmed using dual-energy X-ray absorptiometry (DEXA) measurement [mean *T*-score: − 1.8 (range: − 3.0 to − 0.7)]. Specimen preparation included soft tissue removal and shortening to an equal length of 37 cm below the tip of the greater trochanter. Before cutting the femoral condyles, neck anteversion was recorded for subsequent orientation. Finally, specimens were fixed in a steel cup using Polymethylmethacrylate (Technovit 3040; Heraeus Kulzer, Wehrheim, Germany). The femur was tilted laterally by 8° in the frontal plane and by 6° dorsally in the sagittal plane to simulate single-leg stance and to create bending and torsional moments as previously described [25, 27] (Fig. 2).

**Table 1:** Demographics of donors

Specimen	Side	Age	Height (m)	Weight (kg)	BMI	Sex	*T*-score	Implant	Implant size
1	r	63	1.52	98.9	42.6	f	− 1.5	TwinSys	13
1	l						− 1.9	Optimys	5
2	r	63	1.63	49.9	18.9	f	− 2.1	Optimys	5
2	l						− 2.2	TwinSys	14
3	r	70	1.57	99.8	40.2	f	− 1.2	TwinSys	12
3	l						− 0.7	Optimys	5
4	r	80	1.68	108.9	38.7	f	− 1.5	Optimys	5
4	l						− 1.3	TwinSys	12
5	r	69	1.57	49.9	20.1	f	− 1.4	TwinSys	12
5	l						− 2.3	Optimys	5
6	l	81	1.57	51.7	20.9	f	− 3	TwinSys	14
6	r						− 2.5	Optimys	6

### Implantation and cementation technique

The implantation of the investigated implants was performed alternating, either in the right or the left of six pairs of femurs, by an experienced orthopedic surgeon (TF) according to the manufacturer’s specifications. A third-generation cementing technique was used. A cement restrictor (BonePlug PE, Mathys Ltd., Bettlach, Switzerland) was inserted, to occlude the femoral canal, providing 1 cm distance to the tip of the stem. Before implantation, cleaning of the femoral cavity was performed using a Jet Lavage system (InterPulse, Stryker Corp., USA). It was then thoroughly dried. One unit (40 g) of Palacos R + G bone cement (Heraeus Medical, Hanau, Germany) was vacuum mixed and applied in retrograde fashion via cement gun and pressurized using a femoral seal [28]. The implants were inserted manually and pressure was maintained until the cement was set.

### Measurement of primary stability under dynamic loading

For measurement of relative motion between the implant and the cortical bone, two inductive miniature displacement transducers (HBM WI/5 mm-T; HBM, Darmstadt, Germany) with a precision of 1 µm were attached to the cortical bone. Relative axial implant–bone motion was measured at transducer S1 at the shoulder of the prosthesis (Fig. 2). Rotational stem motion was captured at transducer S2, which was attached perpendicular to the neck of the implant (Fig. 2). The measured micromotions were calculated into rotation around the femoral axis by gauging the distance between the tip of the transducer and the longitudinal axis of the femoral diaphysis [29]. The femur was mounted in a servo hydraulic material-testing machine (Instron, Typ 8871, Pfungstadt, Germany), which applied a vertical load. A ball bearing was attached between the device and the load cell to achieve a moment-free introduction of the load (Fig. 2). The material-testing machine applied 100,000 dynamic sinusoidal load cycles at a frequency of 2 Hz between 100 and 1600 N to simulate the load of the first 6 weeks in vivo [30]. Reversible implant-bone motion was captured every 500 cycles at the two measurement points for all samples. Furthermore, irreversible implant migration in axial direction (S1) was calculated by the displacement between the initial implant position and the position at the end of 100,000 loading cycles. In the same way, irreversible torsion around the femoral axis was calculated from the displacement assessed at transducer S2.

### Assessment of fracture load and fracture pattern

After dynamic loading, repeated radiographs in two planes were performed to exclude periprosthetic fractures of specimens. Subsequently, specimens were transferred to the testing machine and linearly loaded at a rate of 100 N/s under load control until a fracture occurred. The fracture load (Fmax) was assessed and fracture pattern was analyzed using the Vancouver Classification [32].

### Statistical analysis

Statistical analysis was performed using SAS 9.4 software (SAS Institute, Cary, NC). Normality testing indicated that the data were non-parametric in nature and so testing was performed using Wilcoxon signed-rank to analyze differences of reversible and irreversible micromotions as well as of fracture loads between the two implants. Significance was assumed for *p* ≤ 0.05.

## Results

### Reversible micromotion measurement

After 100,000 loading cycles mean micromotion amplitudes at both transducer locations did not display any statistical differences between both femoral implants. Mean axial micromotions were 5.3 µm (SD 3.9 µm) for the short stem in comparison to 9.3 µm (SD 6.6 µm) for the conventional stem (*p* = 0.23). The calculated rotation around the femoral axis was in direction of retroversion for both stems, with values of 0.03° (SD 0.02°) for the short stem and 0.04° (SD 0.02°) for the conventional stem (*p* = 0.44; Table 2).

**Table 2:** Measurements of reversible micromotion and irreversible migration after 100,000 loading cycles up to 1600 N

Implant	Mean	SD	Min	Max
Axial Micromotion (S1) [µm] (*p* = 0.23)				
Optimys	5.3	3.9	2.2	12.8
TwinSys	9.3	6.6	2.8	17.2
Rotational Micromotion (S2) [°] (*p* = 0.44)				
Optimys	0.03	0.02	0.02	0.04
TwinSys	0.04	0.02	0.01	0.07
Axial Migration (S1) [µm] (*p* = 0.22)				
Optimys	− 20.4	38.3	− 92.7	8.7
TwinSys	− 61.4	92.8	− 234.4	25.4
Rotational Migration (S2) [°] (*p* = 0.09)				
Optimys	0.003	0.04	− 0.05	0.04
TwinSys	0.09	0.12	− 0.10	0.24

### Irreversible migration measurement

Mean axial migration after 100,000 loading cycles was − 20.4 µm (SD 38.3 µm) for the short stem and − 61.4 µm (SD 92.8 µm) for the conventional stem (*p* = 0.22). Only minor rotation towards retroversion was measured, with 0.003° (SD 0.04°) for the short stem and 0.09° (SD 0.12°) for the conventional stem (*p* = 0.09) with a tendency towards less retroversion in the group of the short stems (Table 2).

### Maximum fracture load and fracture pattern

Mean maximum fracture load (Fmax) to induce a periprosthetic fracture was 3062 N (SD 332 N) in the short-stem group, whereas Fmax load of 3160 N (SD 544 N) induced a fracture in the conventional stem group (Table 3). No significant differences in Fmax load between the short and conventional stems were found (*p* = 0.84).

**Table 3:** Fracture load (Fmax) inducing a periprosthetic fracture in both stem types

Donor	Optimys		TwinSys	
	Fmax (*N*)	Fracture type	Fmax (*N*)	Fracture type
1	2751	B3	2912	B3
2	2885	B3	2463	C
3	3499	B3	2945	C
4	2730	B3	3076	B3
5	3410	B3	3550	C
6	3099	B3	4012	C
Mean	3062		3160	
SD	332		544	

**Fig. 3:**
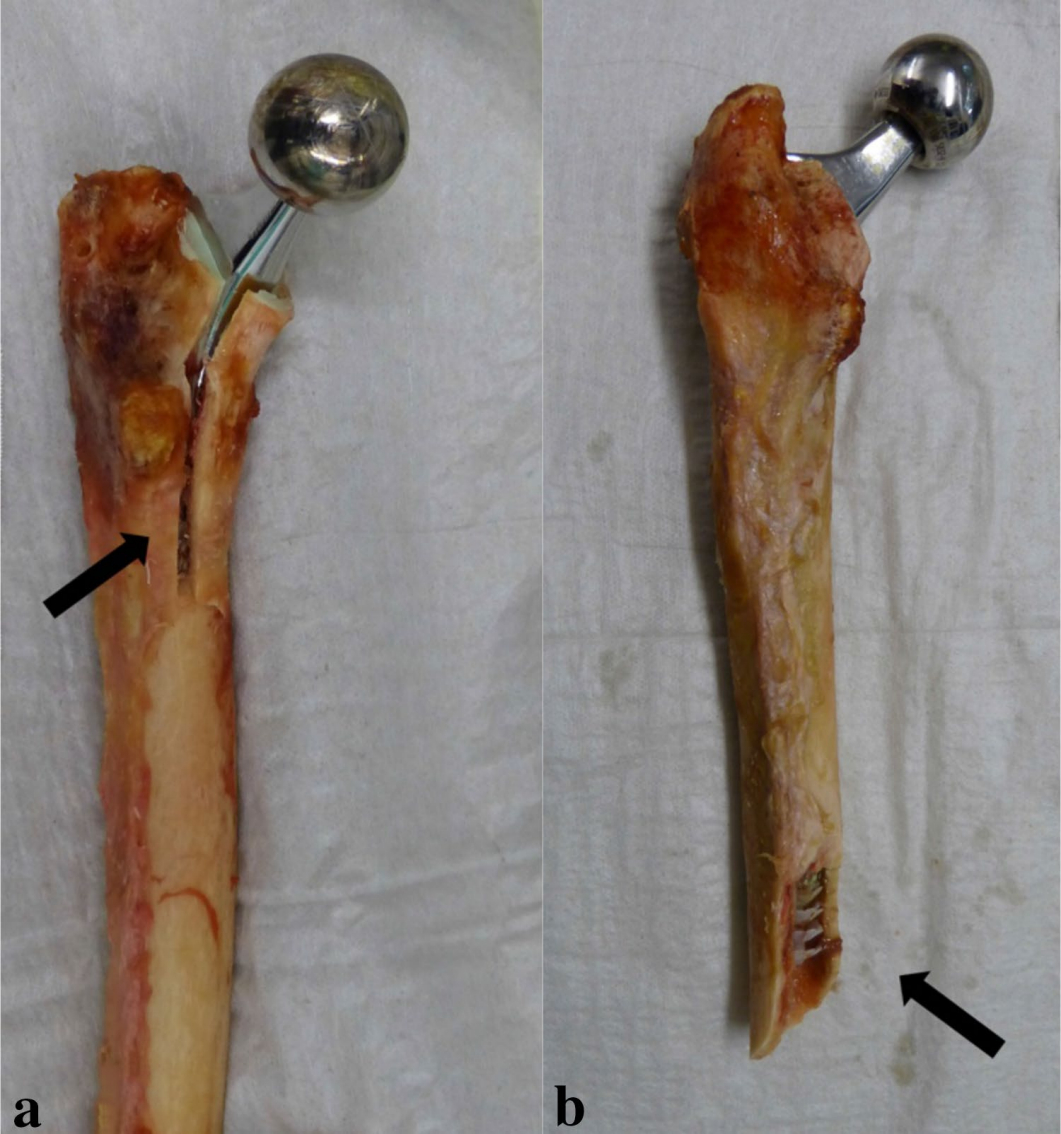
**a**, **b** Fracture pattern of periprosthetic fractures induced in both groups. All short stems showed proximal fractures according to Vancouver type B3 (**a**), 4 out of 6 straight stems showed Vancouver type C fractures (**b**)

**Fig. 4:**
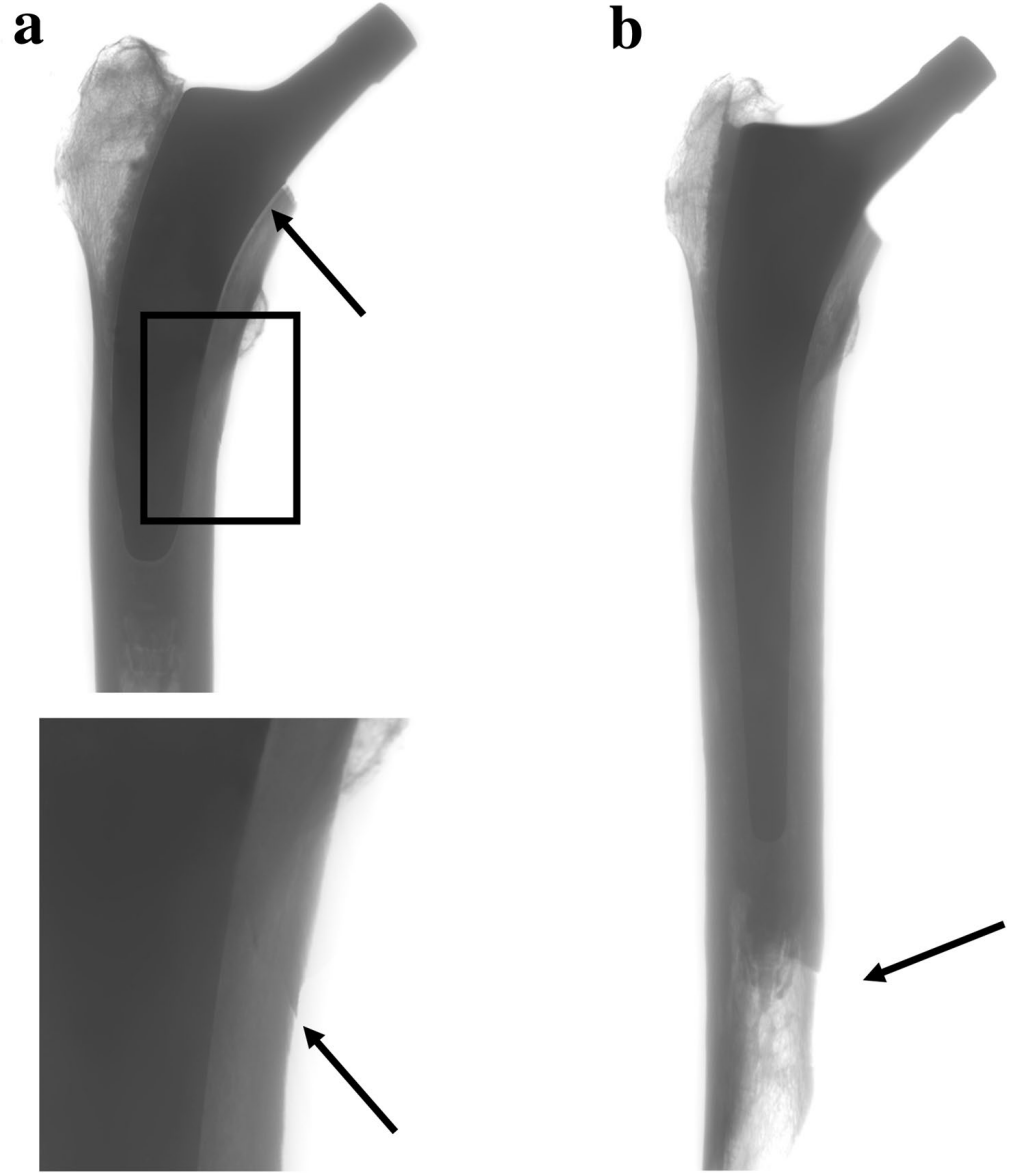
**a**, **b** Anteroposterior radiograph of the periprosthetic femoral fracture with consecutive stem loosening of a cemented short stem (**a**). Anteroposterior radiograph of the fracture of the femur with a cemented conventional stem (**b**)

All short stems fractured under maximum load according to Vancouver type B3 (stem loose, poor bone stock), whereas 4 out of 6 conventional stems suffered a periprosthetic fracture according to type C (well below the tip of the stem). Figures 3, 4a, b exemplify the fracture pattern of periprosthetic fractures induced in both stem designs under controlled conditions.

## Discussion

The aim of this biomechanical in vitro study was to compare the primary stability and maximum fracture load of a newly developed cemented short stem with a clinically proven cemented conventional stem (twinSys) [26]. Our results show that the shorter curved implant design does not negatively influence primary stability and maximum fracture load. However, we found a clear difference in fracture pattern. The short stems fractured according to Vancouver type B3 whereas the conventional stems, except for two preparations, fractured according to type C.

To date, only few studies regarding cemented shorter femoral stems in THA can be found and none, which correspond to the design concept of the present study. Recently, Santori et al. published their 14-year experience with a cemented short stem [31]. However, given that this implant is a derivative of the Exeter straight stem philosophy and has just been shortened, the comparability to the philosophy of new-generation, calcar-guided short stems is limited. The design of the Friendly short stem (LimaCorporate, San Daniele Friuli, Italy) requires the addition of proximal and distal centralizers in the attempt of attaining a 2-mm cement mantle all around the stem. As stated in our recent investigation regarding cementation techniques in contemporary, calcar-guided THA, a line-to-line technique best supports the philosophy of the more individualized implantation technique, compared to most cemented conventional femoral implants [25]. In vitro, it was found to be equivalent to the standard cementation technique using an undersized stem. However, the mid- and long-term results presented by Santori et al. suggest a high reliability of a short, polished and tapered cemented stem without any drawbacks, compared to the conventional sized implants, being obvious [31].

Regarding existing literature involving shorter cemented stems, a second report can be found. Choy et al. presented their experience from the Australian Orthopaedic Association National Joint Replacement Registry (AOANJRR) regarding a 7-year follow-up of Exeter short stems compared to standard-length Exeter stems [32]. No significant difference was found in the cumulative percent revision rate in the short-stem group, compared with the standard-length stems, despite its use in a greater proportion of potentially more difficult hip dysplasia cases. Again, the comparability to the stem design, which was used in the present study, is severely restricted.

The concept of calcar-guided short-stem THA has the potential to preserve bone and soft tissue, by reconstructing the individual anatomy of the patient the best [33]. Furthermore, given a facilitated and less traumatic implantation technique, intraoperative blood loss can be reduced [18]. Recent studies provided beneficial mid-term clinical results of uncemented short stems compared to conventional stem designs along with decreased intraoperative complication rates [19, 20, 34, 35]. Especially neck-sparing short stems seem to have better maintenance of bone mineral density changes compared to conventional implants [35]. Furthermore, there are promising results regarding micromotion in vitro measurements, as well as clinical mid-term results of stem migration patterns and patient-reported outcome measures [24, 36, 37]. It remains unknown, if those potential advantages may be transferred also to the concept of cemented short-stem THA.

The present biomechanical investigation, however, resulted in equivalent results regarding primary stability and maximum fracture load of the newly developed cemented short stem compared to the well-established cemented conventional stem. This is in line with our prior results analyzing the uncemented version of the short stem (optimys), compared to a well-established uncemented conventional stem using the same study protocol [27]. A less pronounced axial and rotational irreversible migration was found for the uncemented short stem, confirming the triple-taper design leads to sufficient stability. In isolated specimens of both stem designs, the measurements showed positive values with regard to migration in axial direction, which in principle would correspond to an implant migration out of the femur. This phenomenon could be explained by a slight tilting of the implant and consecutive elevation of the implant shoulder, on which the displacement transducer was positioned.

In contrast, biomechanical studies of cementless femoral implants showed lower load at failure of shorter stem designs [38, 39]. A cadaver model comparing a double-wedged conventional stem with the Nanos short stem (Smith&Nephew, Marl, Germany) found an increased load at failure for the conventional stem design up to 20% [38]. All specimens of this study suffered a type B2 fracture compromising the medial wall. Gabarre et al. studied the load transmission of the Minihip stem (Corin, Cirencester, United Kingdom) in a finite element model and found a lever effect with high compressive stresses in areas of the stem in contact with bone [40]. This implant follows a neck-sparing concept similar to the uncemented optimys. Lateral loading is also supported by bone mineral density measurements for both implants [41, 42]. This could explain decreased fracture load and the fact, that mainly type b2 fractures were observed. However, even small design differences have significant influence on load transmission [43]. Furthermore, cemented stem fixation significantly influences load transmission of the implant in the proximal femur [44]. A biomechanical investigation of Thomsen et al. compared maximum fracture loads and fracture patterns of cemented and uncemented conventional stems in non-osteoporotic bone [45]. The maximum fracture load was found to be significantly higher for cemented stems. Fracture patterns corresponded to Vancouver type B fractures in uncemented stems and Vancouver type C fractures in cemented stems. In the present study, the Vancouver type C fracture pattern can be confirmed for cemented conventional stems. For the cemented short stem, however, the Vancouver type B3 pattern has to be acknowledged in all cases. A different fracture pattern of cemented femoral stems was observed in a biomechanical sawbone model [46]. Measurements showed a significantly lower torque to failure of a shortened Exeter stem compared to the conventional stem length. The authors conclude that both stems are safe to use as the torque to failure was 7–10 times higher than seen in activities of daily life. Furthermore, the authors observed only Vancouver type B2 fractures in both stem models. However, the test model included a single torsional torque which was applied by a material-testing machine until fracture occurred. A similar test setup with lateral load published by Klasan et al. compared a cemented and cementless double-tapered stem with conventional length in a biomechanical cadaver model [47]. The authors found an increased load-to-failure force by 25% for the cemented version. Similar to the above-mentioned study, they only observed fractures at the stem level for both implants with consecutive stem loosening. Our test setup included combined axial load and torsional torque, produced by tilting the preparations in the frontal (8°) and sagittal (6°) plane, which correspond to the conditions of a single-leg stand [48]. Furthermore, only specimens with reduced bone quality were used. This could explain the different results to our observations.

Some limitations have to be acknowledged. The simulation of the first 6 weeks of loading, only allows conclusions to be drawn about the early stage following implantation. Mid-term and long-term characteristics of cemented short stems most likely can only be obtained in a clinical setup in vivo. Furthermore, in vitro models always simplify in vivo conditions. Muscle forces on the hip joint could not be taken into consideration, resulting only in a “worst case” scenario for proximal loading, however, featuring the advantage of high reproducibility.

## Conclusions

The present in vitro study demonstrates that the concept of a cemented calcar-guided short stem can be further pursued. When comparing the cemented short-stem concept to a well-established cemented conventional stem in the present test setting, no significant differences were found regarding primary stability and fracture load. However, a clear difference in the fracture pattern has to be acknowledged. Further investigations should include a clinical observational study, to confirm the present results under clinical conditions in vivo.
